# University students’ mental health and emotional wellbeing during the COVID-19 pandemic and ensuing lockdown

**DOI:** 10.1177/00812463211012219

**Published:** 2021-06

**Authors:** Maretha Visser, Eloise Law-van Wyk

**Affiliations:** 1 Department of Psychology, University of Pretoria, South Africa; 2 Mastercard Foundation Scholars Program, Department for Education Innovation, University of Pretoria, South Africa

**Keywords:** COVID-19, emotional wellbeing, mental health, South Africa, student population, Keyes’ theory of mental health

## Abstract

The COVID-19 pandemic and ensuing lockdown had a profound effect on human life. This research explores the influence of COVID-19-related experiences on the emotional wellbeing and mental health of South African university students 3 months into the pandemic. Research data were obtained from an online survey completed by 5074 students. Students reported difficulties in coping with psychological challenges during the lockdown: 45.6% and 35.0% reported subjective experiences of anxiety and depression, respectively. Students scored low on the mental health continuum. Hierarchical stepwise multiple regression analyses showed that some different dimensions predicted emotional difficulties or wellbeing and mental health – confirming the two continuum theory of Keyes. Students’ serious discomfort during lockdown, difficulty adjusting academically and feeling socially isolated contributed most to emotional difficulties. Females, students in their early years of study and students residing in informal settlements were most at risk of experiencing emotional difficulties. Mental health was most predicted by students’ hopefulness. Social, academic, spiritual and physical wellbeing and positive coping strategies influenced both emotional difficulties and mental health. The research serves to alert university authorities to students’ emotional wellbeing, especially of first-year students and students with limited resources. The results could assist university psychological services to provide appropriate support services to enhance students’ adjustment and promote their mental health amid a public health crisis.

In response to the declaration of the World Health Organization (WHO) of a global public health emergency following the SARS-CoV-2 outbreak (known as the COVID-19 pandemic), many countries implemented lockdown protocols to curb the spread of the coronavirus. The restrictions imposed had a profound effect on all aspects of social life (Holmes et al., 2020) and significantly impeded economies, countries and communities, and the general and mental health of individuals and families.

Contributing to a multitude of recent literature reviews regarding the psychosocial impact of previous epidemics, Chew et al. (2020) found that fears, anxieties and depression were common psychological symptoms. Reasons for increased anxiety included feelings of vulnerability to the infection, disruptions of routines, uncertainty about employment and finances, and fears for the safety and wellbeing of loved ones (Chew et al., 2020). Similarly, Brooks et al. (2020) reported negative psychological effects such as post-traumatic stress symptoms, confusion and anger. Stress was increased by prolonged quarantine, fear of infection, frustration, boredom, inadequate information and supplies, financial loss and stigma. Research established a link between deteriorating mental health and the COVID-19 pandemic and lockdowns. Cited reasons included increased socioeconomic disparities and job losses (Otu et al., 2020), fears of economic implications (Salari et al., 2020), social isolation and changes in family interactions (Prime et al., 2020).

Students also experienced the pandemic’s impact. Worldwide, many campuses closed, while courses moved to online platforms (Yong, 2020). Ma et al. (2020) found in a cross-sectional, nation-wide study of students in China that acute stress (34.9%), anxiety (21.1%) and depressive symptoms (11.0%) were prevalent during the pandemic. Mental health problems were related to fears of being infected and having decreased social support. Using the 7-item Generalized Anxiety Disorder (GAD-7) scale of Spitzer et al. (2006), Cao et al. (2020) found that 24.9% of college students experienced elevated levels of anxiety because of COVID-19’s impact on their academic activities, daily lives (social distancing) and economic prospects. Living in urban areas, residing with parents and having a steady family income shielded college students from anxiety (Cao et al., 2020). The GAD-7 scores of nursing students in Israel during the third week of the lockdown indicated moderate (42.8%) and severe (13.1%) anxiety (Savitsky et al., 2020), attributable to female respondents experiencing social isolation, economic instability, uncertainty, challenges of remote learning and fears of infection. Likewise, the GAD-7 scores of African students from Nigeria indicated severe (24%), moderate (22%) and mild anxiety (30%) during lockdown (Rakhmanov & Dane, 2020). Female students had significantly higher anxiety scores than their male counterparts.

Considering existing evidence that students’ experiences during the pandemic could influence mental health and emotional wellbeing, the current research explored these indicators among students at a South African university after 3 months of pandemic induced lockdown. We explored the holistic functioning of students and the effects of psychosocial and COVID-19-related experiences on their mental health and emotional wellbeing.

We used Westerhof and Keyes’ (2010) two continua model of mental illness and mental health to conceptualise our terminology. According to this model, mental illness and mental health are not opposites, but rather occur and overlap on separate continua. Levels of mental illness co-exist with levels of mental health, creating different states of subjective wellbeing. In our research, the concept of emotional wellbeing describes the presence or absence of emotional difficulties on the mental illness continuum. Mental health refers to the continuum between flourishing and languishing, that is, between functioning well and experiencing subjective feelings of incompleteness, emptiness, or stagnation. Mental health includes the following three core components: emotional health (happiness and satisfaction), psychological wellbeing (purpose in life, self-realisation) and social wellbeing (being of social value) (Westerhof & Keyes, 2010).

Mental health and emotional wellbeing can be affected by imbalances in multiple human dimensions, particularly in untoward circumstances like the pandemic. Our study used the Wellness Wheel, which recognises eight dimensions of wellness (Albrecht, 2014; Carter & Andersen, 2019), to identify components of holistic functioning of students that could have been affected.

## Method

A cross-sectional online survey was used to explore students’ psychosocial experiences during the pandemic.

### Participants

An electronic message was used to invite all registered students at a large residential university in South Africa (*N* = 48,571) to voluntarily complete an online survey without incurring data costs. A self-selected sample of 5074 students (response rate of 10.4%) completed the survey.

### Instruments

Considering the Wellness Wheel (Carter & Andersen, 2019), we constructed a survey including questions on students’ physical, emotional, social, spiritual, financial, environmental and academic experiences (e.g., living conditions, discomfort during the lockdown, fear of infection, and coping strategies). These closed-ended questions were answered on a 3-point Likert-type scale (almost never, occasionally, nearly every day).

Three existing scales were included in the survey. *The Patient Health Questionnaire for Depression and Anxiety* (*PHQ-4*; Kroenke et al., 2009) was used to assess subjective experiences of depression and anxiety as part of the emotional wellbeing scale. The PHQ-4 consisted of two depression items (PHQ-2) and two anxiety items (GAD-2) – shortened versions of the PHQ-9 and GAD-7, respectively. The 2-item scales have been found to explain 84% of the variance of the longer versions. Both 2-item scales have good internal reliability (>0.80) and construct and clinical validity and have been used widely in research (Krafft et al., 2019; Kroenke et al., 2009). On the PHQ-2 and GAD-2 scales, a score of <2 is the cut-point for identifying possible depressive disorders (with sensitivity of 83%–90%) and generalised anxiety (with sensitivity of 88%), respectively. Clinically, the PHQ-4 is used as an ultra-brief screening tool, not a diagnostic tool (Kroenke et al., 2009).

The *Perceived Hope Scale* (*PHS*; Krafft et al., 2019) assesses hope as a positive expectation about the future which may come into play when people feel unable to cope. The PHS revealed good validity and internal consistency (Cronbach’s alpha 0.87–0.89; Krafft et al., 2019).

The *Mental Health Continuum* (*MHC-SF*) was used to assess mental health as defined by Keyes (2002). This 14-item scale contains three items (from Bradburn’s affect scale) that assess emotional health, six items that measure Ryff’s dimensions of psychological wellbeing (i.e., self-realisation, positive relationships, autonomy, mastery, purpose in life and personal growth), and five items that measure Keyes’ dimensions of social wellbeing (i.e., being of value to society). On a 4-point scale, the MHC-SF measures the frequency of respondents’ experiences of each dimension of mental health. A high score indicates flourishing mental health, whereas a low score indicates languishing mental health (Keyes, 2002). This scale has been used in numerous studies worldwide (Keyes, 2007) and has good internal consistency (>0.80) and validity in the South African context (Keyes et al., 2008).

We constructed scales using items that assess experiences of discomfort during lockdown, positive coping strategies, social connectedness, and emotional, academic and spiritual wellbeing. Every student’s responses were totalled and average scores were calculated per scale (a high score indicating positive wellbeing). The reliability of the scales and examples of items are provided in Table 1.

**Table 1.** Description and reliability of the scales used.

Scale	Number of respondents	Number of items	Scale	Range	Alpha	Examples of items
Discomfort during lockdown	*N* = 4238	10	3-point	0–20	.806	Not in control of what happens; isolated from family or friends
Use of positive coping skills	*N* = 4218	14	3-point	0–28	.771	Tried to find meaning or purpose in the situation; maintained a routine and remained productive
Hope (PHS)	*N* = 3847	8	3-point	0–16	.915	Even in difficult times I am able to remain hopeful; Hope outweighs anxiety
Social connectedness	*N* = 3545	8	3-point	0–16	.771	I felt cut off, isolated, lonely; I felt connected to loved ones
Spiritual wellbeing	*N* = 3268	6	3-point	0–12	.715	I relied on my religion and fellow believers to get through the experience
Academic wellbeing	*N* = 3311	14	3-point	0–28	.905	I had difficulty engaging in self-study; I was motivated and able to focus on my studies
Mental health (MHC-SF)	*N* = 3258	14	4-point	0–42	.910	I have something to contribute to society; I am satisfied with my life
Emotional wellbeing (including PHQ-4)	*N* = 3784	13	3-point	0–26	.901	I am feeling down, depressed and hopeless; I am feeling uncertain, uneasy, stressed.

MHC-SF: Mental Health Continuum; PHQ-4: Patient Health Questionnaire for Depression and Anxiety; PHS: Perceived Hope Scale.

### Procedure

During July 2020, an electronic message was used to invite all students of the university to complete the Qualtrics XM survey online without data costs. This took place during and after their midyear online examination, following 3 months of lockdown and 6 weeks of online classes and tests. Settings allowed students to complete the survey only once.

### Ethical considerations

The research complied with all ethics procedures. Research approval was obtained from the Ethics Committee of the Faculty of Humanities, University of Pretoria (HUM019/0420). The consent form required that respondents were 18 years or above, and completing the survey voluntarily and anonymously. Contact details of three organisations providing online consultation for students in distress were provided. Data were filed password-protected providing only researcher access.

### Data analysis

Following a descriptive analysis, a response rate analysis revealed the representativeness of the sample (see Table 2). Females (who are at higher risk of emotional problems according to Bantjes et al., 2019 and Rakhmanov and Dane, 2020) were over-represented, requiring post-stratification weighting to correct for sample bias (Royal, 2019). Thereafter, we constructed the scales and calculated their reliability. Following bivariate analyses, variables that related significantly to emotional wellbeing and mental health, were entered into hierarchical stepwise multiple regression analyses with emotional wellbeing and mental health as dependent variables. Gender was entered first to control for the effect of gender. Data analysis was conducted using SPSS-26.

**Table 2.** Response rate analysis.

Surveys sent			Responses received			Response rate		
Undergraduate								
Male	Female	Total	Male	Female	Total	Male	Female	Total
15 214 (42%)	20 657 (58%)	35 871 (73.9%)	1 343 (33%)	2 771 (67%)	4 114 (84.7%)	8.8%	13.4%	11.5%
Post graduate								
Male	Female	Total						
5 712 (44%)	6 988 (56%)	12 700 (26.1%)	288 (39%)	458 (61%)	746 (15.3%)	5%	6.6%	5.9%
		48 571			4 860^a^			

a 214 students did not complete these items.

## Results

### Characteristics of respondents

A response rate analysis (see Table 2) showed that undergraduate students (11.5% vs. 5.9% postgraduate students) and female students (13.4% vs. 8.8% male students) were over-represented. To correct for gender bias, we did post-stratification weighting.

The majority of the respondents across faculties were undergraduate (84%) and aged 18–21 years (59.3%). Respondents spent lockdown predominantly in urban suburbs (64.0%) with their families (81.1%). A few stayed on their own (7.2%), some in rural areas (13.5%) or informal settlements (2.2%), where resources were possibly limited (see Table 3).

**Table 3.** Respondents’ demographic details.

	%
Gender	
Male	33.6
Female	66.4
Age	
18–21	59.3
22–25	28.5
25+	12.3
Year level	
First	25.3
Second	25.6
Third	21.3
More than three	11.8
Post-graduate	16.0
Faculty	
Natural sciences	21.7
Engineering, building, IT	20.7
Economic sciences	19.6
Humanities	11.0
Education	10.5
Health sciences	7.9
Law	5.1
Veterinary science	2.5
Theology	1.0
Resided during lockdown	
City centre	5.4
Urban suburb	64.0
Township	15.0
Informal settlement	2.2
Rural area	13.5
With whom they stayed during lockdown	
Parents/family	81.1
Friends/partners	11.7
Alone	7.2

### Reaction to COVID-19 and lockdown

The majority of respondents (72.8%) expressed fear of contracting the virus (20.2% were extremely fearful) or concern about family getting ill. About half of the respondents (52.1%) knew someone who had contracted COVID-19 and 2.5% reported that they had contracted the virus themselves by the time of the survey.

A third of the respondents reported extreme discomfort during lockdown. The discomfort score for the group was 9.7 on a scale of 0 (high discomfort) to 20 (low discomfort) (see Table 6). Aspects experienced as most difficult were academic isolation, not feeling in control, experiencing life as on-hold, being isolated from family and friends and having restricted freedom. Some had difficulty obtaining food, and some fell victim to crime or gender-based violence (see Table 4).

**Table 4.** Reaction to COVID-19 and lockdown.

		%
Fear of COVID-19		
Fear of contracting COVID-19	Extremely fearful	20.2
	Moderately fearful	52.6
	Low/not fearful	27.2
Worried families will contract COVID-19	Extremely worried	50.9
	Moderately worried	38.7
	Low/no worry	10.4
Discomfort during lockdown		
Level of discomfort	Extreme discomfort	33.1
	Moderate discomfort	49.1
	Low/no discomfort	17.8
Extreme difficulty with:	Not able to attend classes	55.4
	Feel vulnerable/not in control	54.3
	Feel life is on-hold	54.2
	Isolated from family/friends	42.7
	Freedom restricted	38.6
	Difficulty obtaining food	12.3
	Victim of crime	5.8
	Victim of gender-based violence	1.6
Coping with challenges of lockdown		
Coping strategies	Seek information	60.2
	Seek emotional support	40.0
	Distraction	36.6
	Rely on religion, spirituality	35.1
	Find meaning, focused on goals	32.7
	Escape reality (sleep, entertainment, substances)	31.0
	Stay productive	30.1
	Help others	28.1
	Tried new things, creativity	24.5

Almost a third of respondents (31.6%) reported significant difficulty coping with the psychological challenges of the lockdown, and for 22.1%, it was a traumatic experience. Respondents reported coping by seeking emotional support, staying productive, practicing religion and spirituality, escaping reality by sleeping excessively, engaging in entertainment or using substances and by obtaining information about staying safe (Table 4). Their positive coping score was low (5.7, *SD* = 2.9) on a scale of 0–28 (see Table 6).

### Aspects of wellbeing

#### Physical wellbeing

The majority of respondents reported that the lockdown hampered their physical health and fitness (e.g., their sleep patterns and diet changed; see Table 5).

**Table 5.** Aspects of students’ wellbeing (selected responses).

	%
Physical wellbeing	
Negative effect on health and fitness	50.8
Positive effect on health and fitness	14.2
Slept more	45.6
Slept less	13.0
Sleep-wake inversion	25.1
Ate more food	37.3
Ate more unhealthy food	19.7
Social connectedness	
Negative effect on social functioning	69.2
Positive effect on social functioning	7.2
Felt lonely and isolated	56.7
Felt breakdown in relationships	31.9
Needed emotional and social support	32.6
Valued electronic connection	59.3
Spiritual wellbeing	
Spiritual functioning affected	46.6
Deeper spiritual connection	34.3
Relied on religion to cope	35.1
Felt removed from spiritual pursuits	19.0
Questioned God	15.2
Financial wellbeing	
Pandemic negatively affected finances	56.9
Depend financially on others	23.5
Concern about future employment	57.1
Academic wellbeing	
Negative effect on academic ability	58.9
Positive effect on academic ability	21.9
Worried cannot complete academic year	49.0
Internal distractions (fear, anxiety)	40.5
Difficulty engaging in self-study	31.5
Difficulty adapting to online study	27.4
Hopefulness	
Feeling hopeful in difficult times	30.8
Hope outweighs anxiety	24.9
Never experience hope	17.0
Emotional wellbeing: negative symptoms (almost every day for past month)	
Depressed feelings	35.0
Little pleasure or interest	27.6
PHQ 2 scale < 2	35.0
Feeling nervous, anxious	45.2
Continuously worrying	38.0
GAD-2 scale < 2	45%
Feeling uncertain, stressed	45.3
Increased irritability, anger, frustration	28.8
Reduced self-confidence	27.4
Thoughts/actions of self-harm	10.3
Thoughts of suicide	5.3
Mental health (almost every day for past month)	
Satisfaction with life	24.5
Ability to manage responsibilities	29.2
Ability to grow and become a better person	24.2
Sense of meaning in life	29.2
Ability to have positive relationships	38.5
Society is not a good place	54.9
Society does not make sense	55.7
Do not belong to society	32.3
Not something to contribute to society	32.3

GAD: Generalised Anxiety Disorder; PHQ: Patient Health Questionnaire for Depression and Anxiety.

#### Social connectedness

The majority of respondents reported a negative effect on their social functioning. Many felt lonely and isolated, but they valued connecting electronically with others (see Table 5). The scale score indicated average social connectedness (8.1 on a scale of 1–16; see Table 6).

**Table 6.** Scale scores.

Scale	Mean [95% CI]	*SD*	Range
Discomfort during lockdown	9.83 [9.67, 9.98]	4.46	0 (high discomfort) to 20 (low discomfort)
Using positive coping strategies	5.73 [5.64, 5.84]	2.93	0 (low use of positive coping) to 28 (high use)
Social connectedness	8.20 [8.08, 8.33]	3.67	0 (disconnected) to 16 (connected)
Spiritual wellbeing	4.47 [4.40, 4.54]	2.04	0 (not well) to 12 (spiritually well)
Academic wellbeing	10.34 [10.19, 10.48]	4.23	0 (not coping academically) to 28 (coping well)
Hopefulness	4.64 [4.53, 4.76]	3.26	0 (low hope) to 16 (high hope)
Anxiety	1.69 [1.65, 1.74]	1.37	0 (high anxiety) to 4 (low anxiety)
Depression	1.91 [1.88, 1.95]	1.24	0 (high depression) to 4 (low depression)
Emotional wellbeing	14.86 [14.70, 15.01]	4.61	0 (emotionally unwell) to 26 (emotionally well)
Mental health	11.24 [10.98, 11.49]	7.37	0 (mental health low) to 42 (mental health high)

CI: confidence interval; *SD*: standard deviation.

#### Spiritual wellbeing

Respondents reported that the pandemic affected their spiritual functioning. About a third experienced a deeper spiritual connection, whereas some felt removed from their spiritual pursuits and some questioned God (see Table 5). The scale score indicated a low level of spiritual wellbeing (4.5 on a scale of 0–12; see Table 6).

#### Financial wellbeing

More than half of the respondents experienced financial losses and increased financially dependency. They were especially worried about increasing economic threats to possible future employment.

#### Academic wellbeing

Although some of the respondents experienced a positive effect, many reported reduced academic ability. They feared not completing the academic year, and some had difficulty engaging in self-study and online learning (see Table 5). Students scored a low average on the academic wellbeing scale (10.3 on a scale of 0–28; see Table 6).

#### Hopefulness

About a third of respondents reported being hopeful in difficult situations, with hope decreasing their anxiety, whereas almost a fifth of the respondents reported hopelessness (see Table 5). The respondents’ level of hope was low (4. 6 on a scale of 0–16; see Table 6).

#### Emotional wellbeing

The majority of students (65.2%) reported that the pandemic restricted their emotional functioning, whereas 7.6% felt more positive and optimistic. Of the respondents, 45.6% reported subjective experiences of anxiety on the GAD-2 scale, and 35.0% reported experiences of depression on the PHQ-2 scale during the month preceding the survey. In total, 10.3% contemplated or engaged in self-harming behaviour and 5.3% reported suicidal ideation. Reported negative emotions are summarised in Table 5.

Respondents’ score on the emotional wellbeing scale, assessing the presence or absence of emotional symptoms, was average (14.76 on a scale of 0–26, see Table 6). (The calculation of the scale was based on frequency of responses – the intensity of the feeling or symptom was not weighed.)

In exploring what affected students’ emotional functioning, we controlled for gender differences by conducting a hierarchical stepwise multiple regression analysis of all the variables in bivariate analyses relating significantly to emotional wellbeing. The best overall model was significant, *F*(8, 3345) = 83.48, *p* = .000, and the combination of variables explained 16.6% of the variance in emotional functioning, representing a medium effect size (Ellis & Steyn, 2003). Several coefficients were significant predictors of emotional wellbeing (see Table 7).

**Table 7.** Coefficients of hierarchal stepwise regression analysis for emotional wellbeing.

Model	Standardised Coefficients	*t*	*p*
	Beta		
(Constant)		26.634	.000
Gender	0.064	4.052	.000
Discomfort during lockdown	0.205	11.515	.000
Academic wellbeing	0.145	8.320	.000
Social connectedness	0.101	5.418	.000
Spiritual wellbeing	0.065	3.927	.000
Negative physical functioning and fitness versus healthy and fit as always	−0.068	−4.060	.000
Year of study	0.054	3.401	.001
Informal settlement versus urban suburb	−0.045	−2.818	.005

Only significant coefficients included.

The analysis showed that gender significantly predicted emotional wellbeing. With gender controlled for, the most important coefficients were level of discomfort, academic wellbeing and social connectedness – variables reflecting effects of the pandemic. The year of study, spiritual wellbeing and positive coping strategies contributed positively to emotional wellbeing, whereas poor health and fitness (relative to no effect on health) and residing in informal settlements (relative to urban suburbs) contributed negatively. These variables explained only some of the variance. Students vulnerable to emotional difficulties thus experienced serious discomfort during the lockdown, had difficulty adjusting academically, felt socially disconnected, experienced relatively poor health and fitness. They were in the early years of study (mostly first-year), students from informal settlements and those who lacked positive coping strategies. Interestingly, field of study, fear of infection, hopefulness and financial concerns did not significantly influence emotional wellbeing during the pandemic.

#### Mental health

The respondents scored low (11.1 on a scale of 0–42, see Table 6) on the mental health scale (MHC-SF), indicating that many of the respondents were languishing rather than flourishing. Respondents expressed some sense of satisfaction with life and personal wellbeing (taking responsibility and regarding life as meaningful) but doubted society and did not feel they belonged or could contribute to society (see Table 5).

In exploring what contributed to students’ mental health, variables that related significantly to mental health in bivariate analyses were entered into a hierarchical stepwise multiple regression analysis. Gender was entered first to control for its effect. The best overall model was significant, *F*(7, 3262) = 610.14, *p* = .000, and the combination of variables explained 56.7% of the variance in mental health. The effect size of this value renders it practically important (Ellis & Steyn, 2003). Several coefficients were significant predictors of mental health (see Table 8).

**Table 8.** Coefficients of hierarchal stepwise regression analysis for mental health.

Model	Standardised Coefficients	*t*	*p*
	Beta		
(Constant)		1.232	.218
Gender	0.001	0.099	.921
Hopefulness	0.432	28.975	.000
Social connectedness	0.177	12.905	.000
Use of positive coping	0.160	11.207	.000
Academic wellbeing	0.129	10.007	.000
Spiritual wellbeing	0.072	5.955	.000
Negative physical functioning and fitness versus healthy and fit as always	−0.068	−5.526	.000

Only significant coefficients included.

According to the data obtained, gender did not predict mental health. Hopefulness, social connectedness, positive coping, academic and spiritual wellbeing (in this order) contributed positively to students’ mental health during the pandemic. Poor health and fitness (relative to no effect on health) contributed negatively to students’ mental health.

## Discussion

This research explored various domains of students’ wellbeing during the pandemic, specifically during the first 3 months of lockdown. In line with other research (Cao et al., 2020; Holmes et al., 2020; Rakhmanov & Dane, 2020; Savitsky et al., 2020; Wang et al., 2020), evidence was found that students experienced emotional and mental health challenges during the pandemic. A third of the respondents had difficulty coping with psychological challenges during the lockdown; 22.1% experienced it as traumatic. The scoring on the PHQ-4, a relatively accurate screening tool for depressive disorder and generalised anxiety (Kroenke et al., 2009), indicated that almost half of the students (45.6%) had subjective experiences of anxiety during the month preceding the completion of the survey, whereas 35.0% had experiences of depression. Research among South African tertiary students before the pandemic (Bantjes et al., 2016, 2019) reported prevalence rates of anxiety and depression much lower than this study. Bantjes et al. (2016) reported moderate to severe symptoms of anxiety (15.5%) and depression (11.2%) over a 2-week period, using Beck Depression and Anxiety Inventories (Beck et al., 1996; Osman et al., 2002). Admittedly, varying sampling and assessment methodologies complicate comparisons. A study among students at the same university (Eloff & Graham, 2020) 1 year earlier reported significantly higher mental health scores than those found in our study (11.24 vs 27.23 on a scale of 0–42). However, the cross-sectional nature of our research prevents increased reports of depression and anxiety and decreased mental health, from simply being ascribed to the pandemic. Comparison with previous research suggests that experiences during the pandemic could have had some effect on the emotional wellbeing and mental health of students.

The results of the study showed that various dimensions set out in the Wellness Wheel influenced the emotional wellbeing and mental health of students. The regression analyses showed that, despite an overlap of core components, different experiences affected emotional wellbeing and mental health – the two interconnected continua defined by Westerhof and Keyes (2010). For example, feelings of serious discomfort, being female, being in early years of study and staying in informal settlements with limited resources were unique predictors of emotional difficulties, whereas hopefulness (a trait-like cognitive and goal-oriented dimension according to Krafft et al., 2019) was the most important and unique predictor of mental health, referring to optimum psychological functioning. Academic, social, spiritual and physical wellbeing and positive coping strategies predicted, in different orders, both emotional difficulties or wellbeing and mental health. These variables predicted a rather large variance of mental health among the students.

As with existing research, our results showed that respondents’ academic concerns (Cao et al., 2020; Rakhmanov & Dane, 2020; Savitsky et al., 2020), social distance and isolation (Cao et al., 2020; Savitsky et al., 2020) and positive coping strategies (Chew et al., 2020; Liang et al., 2020) played crucial roles in the experience of emotional difficulty and mental health during the pandemic. Similarly, respondents’ poorer physical health and fitness contributed negatively to both emotional wellbeing and mental health, as in the results of Wang et al. (2020). A significant body of evidence has demonstrated the positive mental health benefits of physical health and fitness (Archer et al., 2014; Paluska & Schwenk, 2000).

Our research confirmed the finding of Wang et al. (2020) that students in early years of study (especially, first years) experienced more emotional difficulties during lockdown than senior students. Young students may experience negative emotional outcomes during crises (Huang & Zhao, 2020; Rossi et al., 2020) due to under-developed coping strategies to deal with major crises.

Our finding that females were more vulnerable than males to the experience of adverse emotional effects during the pandemic confirms the findings of some existing studies (Rakhmanov & Dane, 2020; Rossi et al., 2020; Savitsky et al., 2020), but not all (Huang & Zhao, 2020).

Respondents who resided in informal settlements and resource-restricted settings (as opposed to suburbia) experienced elevated challenges (e.g., overcrowding, lack of infrastructure to support online learning) resulting in relatively more emotional difficulties. This finding supported that of Cao et al. (2020) among Chinese students.

Contrary to other research findings (Chew et al., 2020; Savitsky et al., 2020), fear of infection showed little relationship to emotional and mental health in our study. Respondents might have considered their risk as low and other pressing concerns could have outweighed fear of infection. Also contrary to other findings (Bhorat et al., 2020; Cao et al., 2020), financial concerns did not contribute significantly to our respondents’ emotional and mental health. They might have had fewer financial responsibilities during lockdown. However, similar to the finding of Salari et al. (2020) and Wang et al. (2020), our respondents expressed concern about the economic implications of the pandemic for their future employment opportunities.

Considering our research results, universities should give special attention and support to high-risk undergraduate students. Holmes et al. (2020) and Marques et al. (2020) drew attention to the growth of mental health needs since the COVID-19 crisis and suggested proactive steps to ‘flatten the mental health need curve’ before demand overwhelms the capacity of available services. This is especially important in a setting such as South Africa where mental health resources are limited (Docrat et al., 2019) and often unaffordable to people most in need. Based on the research findings and Wind et al.’s (2020) comments that the pandemic was the impetus to shifting mental health care services online, we propose that more professional psychological services be available to students online. In addition, online peer counselling and group support should be provided during times of crisis. Psychoeducation on coping strategies, health benefits of physical exercise (together with online physical exercise training programmes) and adequate physical self-care could be offered to students, and wraparound services to promote academic resilience. Faculties could consider providing additional academic support in the form of tutorial videos by lecturers (with question and answer sessions) and asynchronous digital classrooms, allowing for flexibility to the benefit of both lecturers and students (Daniel, 2020). Additional tutors could assist in creating learning communities where senior students could help junior students adapt to online learning and build academic resilience.

Since students who resided in informal settlements during the lockdown experienced more adverse emotional outcomes than did other students, these students could be housed in university residences in times of crisis. This may increase their sense of belonging and moderate negative effects on their emotional wellbeing.

The survey was completed in July 2020, during and after the online examination period. The timing of the survey could thus have influenced the students’ responses negatively. The research aimed to explore the broad experiences of students and did not use standardised assessments of depression and anxiety, thus making it difficult to compare the results with other research results. In addition, the lack of longitudinal data is a limiting factor in making inferences about the impact of experiences during the pandemic on students’ emotional wellbeing and mental health.

## Conclusion

The research highlights the emotional difficulties and low levels of mental health of students after 3 months of lockdown during the COVID-19 pandemic. The experiences that negatively contributed to students’ emotional difficulties and low mental health have been identified and can be addressed. The results could alert university authorities to the importance of students’ emotional wellbeing and mental health and the provision of psychological services and more relevant and appropriate support services to students. Moreover, the research results could serve to elucidate more appropriate steps to safeguard students’ psychological wellbeing during a crisis.
